# Transcatheter aortic valve replacement in patients with severe aortic stenosis reduced the frequency of intradialytic hypotension

**DOI:** 10.1038/s41598-024-57213-9

**Published:** 2024-03-18

**Authors:** Makoto Saigan, Masaki Miyasaka, Tasuku Nagasawa, Masataka Taguri, Natsuko Satomi, Manami Watahiki, Masaki Nakashima, Yusuke Enta, Yusuke Toki, Yoshiko Munehisa, Jun Ito, Yukihiro Hayatsu, Norio Tada

**Affiliations:** 1 Department of Cardiology, Sendai Kousei Hospital, Sendai, Miyagi Japan; 2 Department of Laboratory Medicine, The Jikei University School of Medicine, Tokyo, Japan; 3 Division of Nephrology, Endocrinology and Vascular Medicine, Tohoku University Hospital, Sendai, Japan; 4 Department of Health Data Science, Tokyo Medical University, Tokyo, Japan; 5 Department of Cardiovascular Medicine, School of Medicine, Dokkyo Medical University, Shimotsuga-gun, Tochigi, Japan; 6 Department of Anesthesiology and Intensive Care Unit, Sendai Kousei Hospital, Sendai, Miyagi Japan; 7 Department of Cardiovascular Surgery, Sendai Kousei Hospital, Sendai, Miyagi Japan

**Keywords:** Cardiology, Diseases, Nephrology

## Abstract

Intradialytic hypotension (IDH) is a common complication during hemodialysis that increases cardiovascular morbidity and mortality. Aortic stenosis (AS) is a cause of IDH. Transcatheter aortic valve replacement (TAVR) has become an established treatment for patients with severe AS. However, whether TAVR reduce the frequency of IDH has not been investigated. This study aims to verify the efficacy of TAVR for reduction of the frequency of IDH. Consecutive hemodialysis patients who underwent TAVR at Sendai Kosei Hospital from February 2021 to November 2021 with available records 1 month before and 3 months after TAVR were included in the study. IDH was defined as a decrease in systolic blood pressure by 20 mmHg or a decrease in the mean blood pressure by 10 mmHg associated with hypotensive symptoms or requiring intervention. Patients with ≥ 3 episodes of IDH in ten hemodialysis sessions comprised the IDH group. Overall, 18/41 (43.9%) patients were classified into the IDH group. In ten hemodialysis sessions, IDH events were observed 2.1, 4.3, and 0.4 times in the overall cohort, IDH group, and non-IDH group, respectively. After TAVR, the incidence of IDH decreased from 43.2 to 10.3% (p < 0.0001) and IDH improved significantly in 15 patients in the IDH group. The result suggested that severe AS was the major cause of IDH in this cohort, and TAVR may be an effective treatment option for reduction of the frequency of IDH in patients with severe AS.

## Introduction

The prevalence of kidney failure is 0.07%, corresponding to 5.3 million people worldwide. Meanwhile, the number of patients undergoing maintenance hemodialysis is increasing worldwide, with a rate of 2% per year in Europe and the United States and 4% in Latin America^1^, excess of 10% in Asia^2^. Patients on hemodialysis represent a high-risk population with poor long-term survival and a plethora of comorbidities^3^. Aortic stenosis (AS) is the most frequent valvular heart disease in patients on hemodialysis with an incidence of 25–55%, whereas the prevalence of AS in the general population is 2–4%^4^. The prognosis of patients with symptomatic severe AS is poor irrespective of the presence of maintenance dialysis, with survival reported to be 3.8 years after the onset of angina, 2.3 years after the onset of syncope, and 0.9 years after the onset of heart failure^5^; thus, aortic valve replacement is necessary for these patients. Although surgical aortic valve replacement (SAVR) was previously the only available option for severe AS, transcatheter aortic valve replacement (TAVR) has become an alternative treatment option^6–13^. For patients on hemodialysis, TAVR is an important alternative strategy as SAVR is less likely to be offered to these patients due to the perceived increased morbidity and mortality following SAVR^14–17^.

Intradialytic hypotension (IDH) is one of the most common complications during hemodialysis, and its prevalence ranges from 8 to 40%^18–21^. IDH is also reportedly associated with higher mortality^22–24^. IDH is the result of interactions between the degree of ultrafiltration, cardiac output, and arteriolar tone^25^. Therefore, severe AS, which reduces cardiac output, is one of the causes of IDH^26^. There is a clinical impression that AS is likely to be involved in the pathogenesis, and patients with severe AS who experience IDH have a poor prognosis. However, there are no previous studies investigating the efficacy of SAVR or TAVR, which are treatments for severe AS, for reducing the frequency of IDH. The purpose of this study is to investigate whether TAVR effectively reduce the frequency of IDH in patients with severe AS.

## Results

### Baseline characteristics of the study population

**Figure 1:**
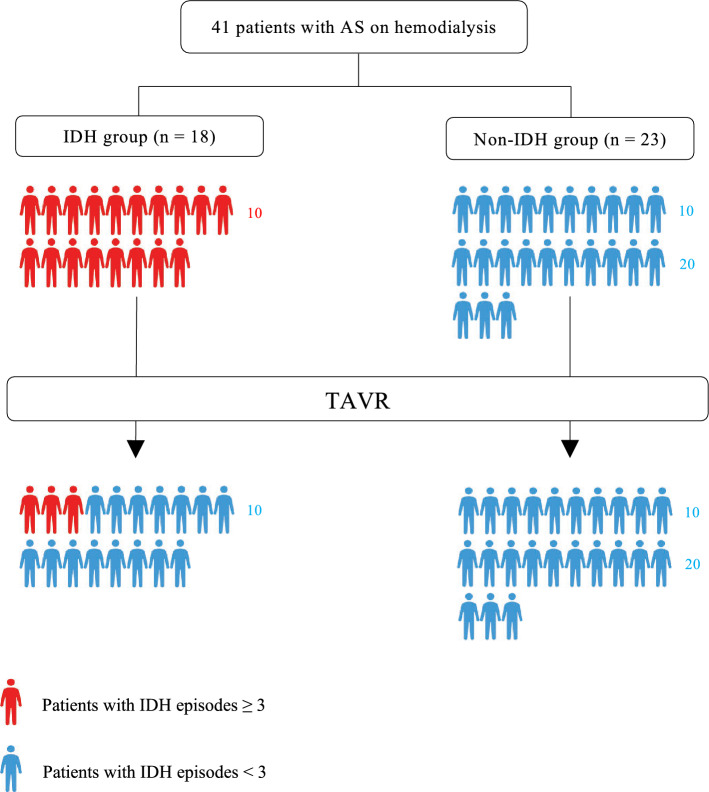
Patient flow through this study. Patients who experienced IDH ≥ 3 times in ten dialysis sessions before and after TAVR. IDH, indicates intradialytic hypotension; TAVR, transcatheter aortic valve replacement.

Of the 41 patients in the study, 18 experienced IDH before TAVR; these patients comprised the IDH group (Fig. 1). The baseline characteristics of these patients are summarized in Table 1. Dyslipidemia tended to be more common in the non-IDH group than in the IDH group. In the other variables, including presence of cardiovascular disease, atrial fibrillation and hemodialysis treatment history, there were no differences between the groups. The use of calcium channel blockers was numerically less frequent in the IDH group. There were no significant differences in the use of other antihypertensive medications between the two groups. In the baseline echocardiographic variables, the non-IDH group had lower indexed aortic valve area and less frequent moderate mitral valve regurgitation (MR). No significant difference in ejection fraction was observed between the groups.

**Table 1:** Baseline characteristics. Values are median (interquartile range) or n (%).

	IDH (N = 18)	non-IDH (N = 23)	p value
Age, years	76.5 (73–83)	79.0 (73–83)	0.85
Female, n (%)	9 (50)	5 (22)	0.06
Height, cm	157 (151–160)	160 (153–165)	0.14
Body weight at hospitalization, kg	52.2 (45.6–59.3)	51.4 (48.6–62.6)	0.50
Body mass index, kg/m^2^	20.8 (19.5–23.2)	19.6 (19.0–23.7)	0.54
BSA, m^2^	1.52 (1.41–1.60)	1.55 (1.40–1.68)	0.37
Preoperative DW, kg	51.0 (43.9–56.9)	50.1 (47.4–61.4)	0.30
Vintage, years	11 (7.8–22.5)	9 (6.0–16.0)	0.89
Diabetic kidney disease, n (%)	4 (22)	9 (39)	0.32
Blood access type, n (%)			0.49
Graft	1 (6)	2 (9)	
Arteriovenous fistula	16 (89)	21 (91)	
Catheter	1 (6)	0 (0)	
spKt/V	1.63 (1.42–1.74)	1.54 (1.25–1.77)	0.49
Ultrafiltration (L)	2.6 (1.8–2.9)	2.4(2.2–2.8)	0.96
Treatment time delivered (min)	240 (240–248)	230 (200–250)	0.14
Symptom during hemodialysis, n (%)	12(67)	2(9)	0.0002
Intervention during hemodialysis, n (%)	18(100)	4(17)	< 0.0001
Comorbidities			
Hypertension, n (%)	13 (72)	19 (83)	0.43
Dyslipidemia, n (%)	7 (39)	18 (78)	0.01
Diabetes mellitus, n (%)	4 (22)	9 (39)	0.25
Atrial fibrillation, n (%)	7 (39)	5 (22)	0.23
COPD, n (%)	1 (6)	3 (14)	0.31
Current smoker, n (%)	0 (0)	2 (9)	0.50
NYHA functional class, III or IV, n (%)	8 (44)	10 (43)	0.47
Prior MI, n (%)	1 (6)	4 (17)	0.36
Prior PCI, n (%)	6 (33)	9 (39)	0.75
Prior CVA, n (%)	2 (11)	3 (14)	0.81
Prior CABG, n (%)	1 (6)	1 (4)	1.00
Prior CAD, n (%)	7 (39)	9 (39)	1.00
Prior PAD, n (%)	3 (17)	4 (17)	1.00
Previous device implantation			
Pacemaker, n (%)	4 (22)	1 (4)	0.15
ICD, n (%)	0 (0)	0 (0)	1.00
CRT or CRTD, n (%)	0 (0)	0 (0)	1.00
Logistic EuroSCORE, %	3.9 (2.4–7.0)	4.7 (1.9–7.7)	0.71
STS score, %	12.9 (12.0–19.5)	14.4 (6.6–19.3)	0.96
Serum laboratory values			
Albumin, g/dL	3.8 (3.2–4.0)	3.5 (3.3–3.8)	0.50
WBC, × 103/mm^3^	50 (34–75)	47 (34–62)	0.45
Hemoglobin, g/dL	11.5 (10.4–12.6)	11 (10.1–12.6)	0.62
Phosphorus , mg/dL	5.4 (4–6.1)	4.9 (4.3–6.1)	0.74
Calcium, mg/dL	9.0 (8.3–9.5)	8.7 (8.5–9.3)	0.97
I-PTH, pg/mL	121 (47–168)	116 (39–184)	0.99
Creatinine, g/dL	5.9 (4.7–7.1)	6.4 (5.1–7.3)	0.28
BUN, mg/dL	29.8 (20.9–41.6)	34.0 (29.2–1.5)	0.27
BNP, pg/mL	894 (595–3,900)	1897 (716–5,238)	0.23
Drug			
β–blocker use, n (%)	12 (66.7)	9 (39)	0.08
Calcium channel blocker use, n (%)	7 (38.9)	16 (70)	0.05
Renin–angiotensin system blocker use, n (%)	5 (27.8)	10 (43)	0.30
Midodrine use, n (%)	0 (0)	0 (0)	1.00
Echocardiographic data			
AVA, cm^2^	0.82 (0.70–0.89)	0.70 (0.61–0.86)	0.20
Indexed AVA, cm^2^/m^2^	0.53 (0.39–0.57)	0.45 (0.36–0.51)	0.05
Peak velocity, m/s	4.08 (3.56–4.65)	4.45 (3.91–5.04)	0.17
Mean gradient, mmHg	42 (32–47)	56 (38–62)	0.08
LVEF, %	51 (43–56)	49 (38–57)	0.78
SV, mL	53 (50–71)	67 (47–76)	0.17
SVI, ml/m^2^	36 (31–47)	42 (31–50)	0.49
AR ≥ moderate, n (%)	1 (6)	2 (9)	1.00
MR ≥ moderate, n (%)	4 (22)	0 (0)	0.03
MS ≥ moderate, n (%)	0 (0)	0 (0)	1.00

*AR* Aortic valve regurgitation, *AVA* aortic valve area, *BNP* Brain natriuretic hormone, *BSA* Body surface area, *BUN* Blood urea nitrogen, *CABG* Coronary artery bypass grafting, *CAD* Coronary artery disease, *COPD* Chronic obstructive pulmonary disease, *CRT* Cardiac resynchronization therapy, *CRTD* Cardiac resynchronization therapy defibrillator, CVA Cerebrovascular accident; *DW* dry weight, *EuroSCORE* European system for cardiac operative risk evaluation, *ICD* Implantable cardioverter defibrillator, *IDH* Intradialytic hypotension, *I-PTH* Intact-parathyroid hormone, *LVEF* Left ventricular ejection fraction; *MI* Myocardial infarction, *MR* Mitral valve regurgitation, *MS* Mitral valve stenosis, *NYHA* New York heart association, *PAD* Peripheral artery disease, *PCI* Percutaneous coronary intervention; *STS* Society of thoracic surgeons, *SV* Stroke volume, *SVI* Stroke volume index, *WBC* White blood cells.

### Procedural characteristics and outcomes and post-procedural echocardiographic date

The procedural characteristics and outcomes are listed in Table 2, and the post-procedural echocardiographic data are listed in Table 3. There were no differences in procedure-related aspects, prosthetic valve function-related variables and the incidence of at least moderate paravalvular aortic regurgitation between the two groups. Meanwhile, more patients had at least moderate MR in the IDH group.

**Table 2:** Procedural characteristics and outcomes.

	IDH (N = 18)	non-IDH (N = 23)	p value
Procedural Characteristics			
Approach			0.69
Transfemoral, n (%)	15 (83)	21 (91)	
Transsubclavian, n (%)	2 (11)	1 (4)	
Transaortic, n (%)	1 (6)	1 (4)	
Balloon pre-dilatation, n (%)	4 (22)	1 (4)	0.16
Balloon post-dilatation, n (%)	9 (50)	16 (70)	0.33
Contrast volume, mL	110 (80–152)	116 (98–142)	0.39
Procedure time, min	634 (44–98)	54 (44–74)	0.75
Fluoroscopy time, min	26 (15–37)	21 (18–31)	0.91
Procedure Outcomes			
Procedure success, n (%)	18 (100)	23 (100)	1.00
Coronary obstruction, n (%)	0 (0)	0 (0)	1.00
Percutaneous cardiopulmonary bypass, n (%)	0 (0)	0 (0)	1.00
Conversion to open–heart surgery, n (%)	0 (0)	0 (0)	1.00
Valve–in–valve, n (%)	0 (0)	0 (0)	1.00
Pericardial tamponade, n (%)	0 (0)	0 (0)	1.00
Vascular complications, n (%)	2 (11)	0 (0)	0.19
Stroke, n (%)	0 (0)	1 (4)	0.56
Life threatening Bleeding, n (%)	1 (6)	0 (0)	0.44
Need for blood transfusion, n (%)	4 (22)	5 (22)	0.97
Myocardial infarction, n (%)	0 (0)	0 (0)	1.00
New pacemaker, n (%)	1 (6)	2 (9)	1.00
New–onset atrial fibrillation, n (%)	0 (0)	0 (0)	1.00
Length of stay, days	15 (13–19)	16 (10–25)	0.80
Discharge to home, n (%)	15 (83)	19 (83)	0.95

Values are median (interquartile range) or n (%). Abbreviations as in Table 1.

**Table 3:** Post-procedural echocardiographic data.

	IDH (N = 18)	non-IDH (N = 23)	p value
EOA, cm^2^	1.73 (1.50–1.88)	1.74 (1.52–1.98)	0.86
Indexed EOA, cm^2^/m^2^	1.08 (1.03–1.35)	1.12 (1.03–1.26)	0.98
Peak velocity, m/s	2.24 (2.04–2.83)	2.68 (2.14–2.86)	0.38
Mean gradient, mmHg	11 (9–17)	18 (11–18)	0.33
LVEF, %	52 (39–61)	49 (42–54)	0.38
SV, mL	62 (51–73)	72 (58–86)	0.10
SVI, ml/m^2^	39 (35–51)	47 (36–54)	0.22
Paravalvular AR ≥ moderate, n (%)	0 (0)	0 (0)	1.00
MR ≥ moderate, n (%)	4 (22)	0 (0)	0.03

Values are median (interquartile range) or n (%).

*EOA* effective orifice area, other abbreviations as in Table 1.

### Incidence of IDH events and blood pressure during dialysis

On average, IDH occurred 2.1 times in ten dialysis sessions (21.4%) in the entire cohort. In the IDH group, 15 of 18 patients had reduced occurrence of IDH after TAVR (Fig. 1). BP during dialysis and incidence of IDH events are listed in Table 4. The incidence of IDH significantly improved from 43.2% to 10.3% before and after TAVR in the IDH group, respectively (p < 0.0001). In contrast, there was no change in the incidence of IDH in the non-IDH group (4.33 to 3.24%; p = 0.70). Additionally, the nadir BP (systolic, mean, and diastolic) and BP variability (systolic, diastolic) during dialysis in the IDH group improved after TAVR (Table 4).

**Table 4:** Blood pressure during dialysis and incidence of IDH events.

	IDH group (N = 18)			non-IDH group (N = 23)		
	pre-TAVR	post-TAVR	p value	pre-TAVR	post-TAVR	p value
IDH events						
Incidence of IDH events per 10 dialysis sessions, (%)	4.32 (43.2)	1.03 (10.3)	< 0.0001	0.43 (4.33)	0.32 (3.24)	0.70
Systolic BP, mmHg						
Pre-HD BP (A)	138 (119–149)	143 (124–160)	0.19	145 (130–174)	160 (140–173)	0.33
Nadir BP during HD (B)	80 (74–95)	101 (94–112)	0.0002	116 (95–135)	115 (100–140)	0.87
Δintradialytic BP (A) − (B)	53 (29–62)	32 (21–46)	0.003	21 (15–45)	38 (20–56)	0.23
Post-HD BP	115 (108–127)	132 (117–164)	0.01	150 (130–157)	144 (135–163)	0.68
Mean BP, mmHg						
Pre-HD BP (C)	91 (81–98)	97 (80–107)	0.34	95 (87–107)	94 (90–105)	0.65
Nadir BP during HD (D)	64 (51–71)	75 (67–85)	0.004	79 (66–90)	82 (65–93)	0.86
Δintradialytic BP (C) − (D)	25 (13–39)	17 (7–27)	0.03	12 (8–19)	17 (9–25)	0.47
Post-HD BP	83 (73–93)	92 (82–98)	0.08	94 (86–104)	87 (81–99)	0.39
Diastolic BP, mmHg						
Pre-HD BP (E)	65 (60–73)	66 (56–85)	0.29	73 (66–80)	67 (60–75)	0.98
Nadir BP during HD (F)	47 (37–54)	53 (45–65)	0.01	60 (49–70)	55 (49–69)	0.86
Δintradialytic BP (E) − (F)	19 (11–36)	8 (3–25)	0.07	10 (5–20)	10 (1–17)	0.21
Post-HD BP	64 (58–73)	74 (59–77)	0.10	70 (60–72)	70 (54–80)	0.89

Values are median (interquartile range) or n (%).

*BP* Blood pressure, *HD* Hemodialysis, *TAVR* Transcatheter aortic valve replacement, other abbreviations as in Table 1.

**Figure 2:**
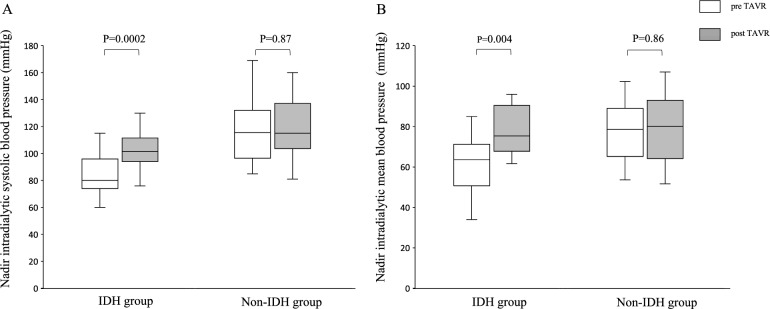
Nadir intradialytic blood pressure before and after TAVR in the IDH and non-IDH group. Nadir intradialytic systolic (**A**) and mean (**B**) blood pressure. IDH, indicates intradialytic hypotension; TAVR, transcatheter aortic valve replacement.

Figure 2 shows the nadir BP during dialysis before and after TAVR in both groups. In the IDH group, the nadir BP during dialysis was higher after TAVR than before TAVR (Figs. 2 A,B). In contrast, the nadir BP during dialysis between before and after TAVR was not different in the non-IDH group (Figs. 2 A,B).

### Dry weight and echocardiographic data pre- and post-TAVR

Preoperative dry weight (DW) was not significantly different between the two groups (Table 1). Postoperative DW was also not significantly different between the IDH and the non-IDH group (48.52 vs. 48.54 kg; p = 0.20). In both the IDH and non-IDH groups, DW was significantly lower after TAVR than before TAVR (Table 5). Echocardiography showed that the effective orifice area increased and both peak velocity and mean gradient decreased after TAVR compared to before TAVR in both the IDH and non-IDH groups, whereas stroke volume (SV) and SV index (SVI) increased numerically but did not change significantly (Table 5).

**Table 5:** Dry weight and echocardiographic data pre- and post-TAVR.

	IDH group (N = 18)			non-IDH group (N = 23)		
	pre-TAVR	post-TAVR	p value	pre-TAVR	post-TAVR	p value
DW, kg	51.0 (43.9–56.9)	48.5 (43.0–55.8)	0.004	50.1 (47.4–61.4)	48.5 (44.7–60.1)	0.01
EOA, cm2	0.82 (0.70–0.89)	1.73 (1.50–1.88)	< 0.0001	0.70 (0.61–0.86)	1.74 (1.52–1.98)	< 0.0001
Indexed EOA, cm2/m2	0.53 (0.39–0.57)	1.08 (1.03–1.35)	< 0.0001	0.45 (0.36–0.51)	1.12 (1.03–1.26)	< 0.0001
Peak velocity, m/s	4.08 (3.56–4.65)	2.24 (2.04–2.83)	< 0.0001	4.45 (3.91–5.04)	2.68 (2.14–2.86)	0.0001
Mean gradient, mmHg	42 (32–47)	11 (9–17)	0.0001	56 (38–62)	18 (11–18)	0.0001
LVEF, %	51 (43–56)	52 (39–61)	0.27	49 (38–57)	49 (42–54)	0.60
SV, mL	53 (50–71)	62 (51–73)	0.61	67 (43–76)	72 (58–86)	0.07
SVI, ml/m2	36 (31–47)	39 (35–51)	0.56	42 (31–50)	47 (36–54)	0.07

Values are median (interquartile range) or n (%). Abbreviations as in Table 1,3 and 4.

## Discussion

This study revealed two important clinical findings. First, 43.9% of the patients on maintenance dialysis who underwent TAVR experienced IDH. Second, the nadir BP increased and BP variability decreased during hemodialysis in the IDH group after TAVR. Accordingly, the incidence of IDH after TAVR significantly improved from 43.2 to 10.3% in the IDH group.

Although IDH is associated with cardiovascular morbidity and mortality^22–24^ and AS is one of the causes of IDH^26^, little information is available on the frequency of IDH in patients with severe AS and the impact of AS on IDH. Furthermore, it remains unknown whether SAVR or TAVR is an effective treatment for decreasing the frequency of IDH in patients with severe AS. In our study, 43.9% of patients experienced IDH preoperatively, and the frequency of IDH decreased significantly from 43.2 to 10.3% after TAVR (Table 4). These results suggested that one of the most likely causes of IDH in this cohort was AS. Therefore, it is important for clinicians, especially those involved in dialysis treatment, to recognize that AS is a cause of IDH, and that AS can be treated by TAVR.

**Figure 3:**
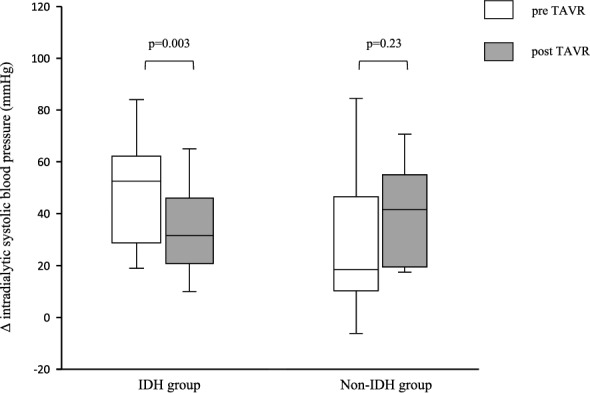
Δintradialytic systolic blood pressure before and after TAVR in the IDH and non-IDH group. Δintradialytic systolic blood pressure was defined as pre-hemodialysis blood pressure minus nadir blood pressure during hemodialysis. Δintradialytic systolic blood pressure represents the variability of systolic blood pressure during hemodialysis. IDH, indicates intradialytic hypotension; TAVR, transcatheter aortic valve replacement.

The increase in nadir BP and decrease in BP variability during hemodialysis after TAVR indicates that TAVR stabilized the BP during dialysis, thereby reducing the frequency of IDH in the IDH group. As for the nadir BP, a significant increase in the systolic, mean, and diastolic BPs during dialysis after TAVR was observed in the IDH group, whereas those in the non-IDH group were not different from before TAVR (Fig. 2 and Table 4). The BP variability in Fig. 3 and data in Table 4 show that the IDH group had smaller BP variability during hemodialysis after TAVR than before TAVR, especially in the systolic and mean BPs. Meanwhile, no consistent changes were observed in the non-IDH group (Fig. 3). Stabilizing the BP during hemodialysis reduces the need for interventions such as intravenous fluid administration, medication, and dialysis discontinuation. BP stabilization also reduces patient’s symptoms and allows for the completion of hemodialysis without the need for additional medications.

Hemodialysis patients often face IDH due to the challenge of achieving euvolemia through ultrafiltration. BP is regulated by various mechanisms, including cardiac output and total peripheral resistance. In hemodialysis patients, the regulatory mechanisms often fail, leading to IDH, which is influenced by several factors, including cardiac output (dependent upon preload, afterload, heart rate, and contractility), arteriolar vasoconstriction, autonomic nervous system activity, vasopressor hormones, and plasma refill^25,27^. In this study, DW, which is an index reflecting preload, was set lower postoperatively in both the IDH and non-IDH groups, suggesting that there was fluid retention before TAVR. The removal of AS may have alleviated fluid retention without causing IDH. We were unable to conduct a detailed examination of vasopressor hormones or the autonomic nervous system. While the frequency of diabetes mellitus (DM) related neuropathy was not extensively confirmed, the frequency of DM and DM-related nephropathy did not significantly differ between the two groups. SV and SVI increased numerically but did not change significantly after TAVR compared to before TAVR in both IDH and non-IDH groups (Table 5), as a previous study showed that cardiac output indices, such as SV and SVI did not change significantly before and after TAVR^28^. Although, it is challenging to establish the exact cause of the decline in the incidence of IDH after TAVR in this study, the removal of the outflow obstruction due to AS may have improved cardiac reserve and could have contributed to the reduction in the frequency of IDH.

### Limitations

Our data should be interpreted in light of the limitations of this study. First, this is a nonrandomized, observational, single-center study. Second, the limited number of cases precluded the possibility of conducting multivariate analysis. Third, some details of the dialysis conditions have not been identified. Fourth, in this study, only TAVR was performed as treatment for severe AS. Therefore, the efficacy of SAVR for reduction of the frequency of IDH in patients with severe AS was not evaluated.

## Conclusions

Among patients on hemodialysis who underwent TAVR, IDH occurred in 43.9%. After TAVR for AS in patients who experienced IDH before the procedure, incidence of IDH decreased from 43.2 to 10.3%, suggesting that the cause of IDH in this study was at least in part severe AS. TAVR may be an effective treatment option for reduction of the frequency of IDH in patients with severe AS.

## Methods

### Study population

Overall, 47 consecutive patients on hemodialysis with severe AS who underwent TAVR at Sendai Kosei Hospital from February 2021 to November 2021 were identified. Dialysis records of the patients 1 month before and 3 months after TAVR were collected. Six patients with missing records due to death from periprocedural complications (n = 4) and absence of records (n = 2) were excluded from the study. Hence, 41 patients were included in the final analysis. This study was conducted in accordance with the Declaration of Helsinki and was approved by our institutional ethics committee. Written informed consent was obtained from all patients.

### Procedures

We performed TAVR using SAPINE3, including non-femoral approaches. In Japan, the use of self-expandable valves for dialysis patients has not been approved, and only the Edwards Sapien 3 (Edwards Lifesciences, Irvine, California) balloon-expandable valve was used. All procedures were performed under general anesthesia guided by transesophageal echocardiography in a hybrid operating room.

### IDH definition

The definition of IDH differs slightly across various guidelines and literature. Differences are found in the blood pressure (BP) parameters, such as the decrease in systolic BP (SBP), nadir SBP, or decrease in mean arterial pressure (MAP), the cut-off value for BP parameters, and symptoms and/or need for intervention^29–34^. In this study, the presence of IDH was confirmed if the following criteria were met:

1. Hypotension, which was defined as a decrease in either the SBP by ≥ 20 mmHg or in the MAP by ≥ 10 mmHg, according to previous studies^32,33^.
2. Symptomatic hypotension or hypotension requiring intervention, including discontinuation of dialysis, inotropic agents, and elevation of the lower extremities.

Patients with ≥ 3 episodes of IDH in 10 hemodialysis sessions comprised the IDH group^35,36^.

### Statistical analysis

All statistical analyses were conducted using JMP 12.1.0. Software (SAS Institute, Inc., Cary, NC, USA). Continuous variables are presented as medians and interquartile ranges. The Mann–Whitney U test was used to assess for significant differences in continuous variables. When comparing time-series data, such as BP, before and after TAVR, the Wilcoxon signed-rank test was used. The Chi-square test or Fisher exact test were used to compare qualitative variables. All analyses were considered statistically significant at a two-tailed p value < 0.05.

### Ethical statement

Our registry was approved by the local Ethical Committee at the Sendai Kousei Hospital in accordance with the Declaration of Helsinki on October 28, 2021 (IRB Number 4–43). Informed consent was obtained from all participants after receiving a full written and oral explanation of the purpose of our registry.

## Data Availability

The datasets analyzed during the current study are not publicly available due to internal procedures but are available from the corresponding author on reasonable request.
