# Author Correction: Transcatheter aortic valve replacement in patients with severe aortic stenosis reduced the frequency of intradialytic hypotension

**DOI:** 10.1038/s41598-024-63612-9

**Published:** 2024-06-05

**Authors:** Makoto Saigan, Masaki Miyasaka, Tasuku Nagasawa, Masataka Taguri, Natsuko Satomi, Manami Watahiki, Masaki Nakashima, Yusuke Enta, Yusuke Toki, Yoshiko Munehisa, Jun Ito, Yukihiro Hayatsu, Norio Tada

**Affiliations:** 1 Department of Cardiology, Sendai Kousei Hospital, Sendai, Miyagi Japan; 2 Department of Laboratory Medicine, The Jikei University School of Medicine, Tokyo, Japan; 3 Division of Nephrology, Endocrinology and Vascular Medicine, Tohoku University Hospital, Sendai, Japan; 4 Department of Health Data Science, Tokyo Medical University, Tokyo, Japan; 5 Department of Cardiovascular Medicine, School of Medicine, Dokkyo Medical University, Shimotsuga-gun, Tochigi, Japan; 6 Department of Anesthesiology and Intensive Care Unit, Sendai Kousei Hospital, Sendai, Miyagi Japan; 7 Department of Cardiovascular Surgery, Sendai Kousei Hospital, Sendai, Miyagi Japan

Correction to: *Scientific reports* 10.1038/s41598-024-57213-9, published online 18 March 2024

In the original version of this Article Makoto Saigan and Masaki Miyasaka were omitted as equally contributing authors.

The original Article has been corrected.

